# Aberrant ontogeneses and life cycles in Paraneoptera

**DOI:** 10.3897/compcytogen.v15.i3.70362

**Published:** 2021-08-25

**Authors:** Ilya A. Gavrilov-Zimin

**Affiliations:** 1 S.I. Vavilov Institute for the History of Science and Technology of the Russian Academy of Sciences, Universitetskaya nab. 5, St. Petersburg, 199034, Russia S.I. Vavilov Institute for the History of Science and Technology of the Russian Academy of Sciences St. Petersburg Russia

**Keywords:** Exuviatrium, imago, larvae, metamorphosis, nymphs, protoptera, pseudopuparium

## Abstract

The paper is a third part of the themed issue “Aberrant cytogenetic and reproductive patterns in the evolution of Paraneoptera”, prepared by a Russian-Bulgarian research team on the basis of long-term collaborative studies. This chapter reviews different peculiar aberrations in the ontogenesis of Paraneoptera, such as the appearance of the quiescent apodal and/or arostrate instars, exuviatrial, pupillarial and pseudopupillarial development, cyclic parthenogenesis, etc. The material and methods, terminology and the nomenclature of the used taxonomic names are listed in the first chapter of the issue (Gavrilov-Zimin et al. 2021).

The postembryonic ontogenesis of most Paraneoptera exhibits simple direct development from primolarva to imago and includes 5–6 immature instars in both sexes (see, for example, Poisson and Pesson 1951; Zakhvatkin 1975; Štys and Davidova-Vilimova 1989; Gavrilov-Zimin 2018; Kluge 2020) with the presence of protoptera (wing buds) in instars 3–5(6), which are named “nymphs” in contrast to first larval instars lacking protoptera (Fig. 1). All postembryonic instars of such ontogenesis are actively mobile and feeding. This type of development is undoubtedly a plesiomorphic, archaic condition, inherited by Copeognatha (Psocoptera) (Fig. 2) from the common ancestor of all Paraneoptera and shared with the most other “hemimetabolous” insects.

**Figure 1.**
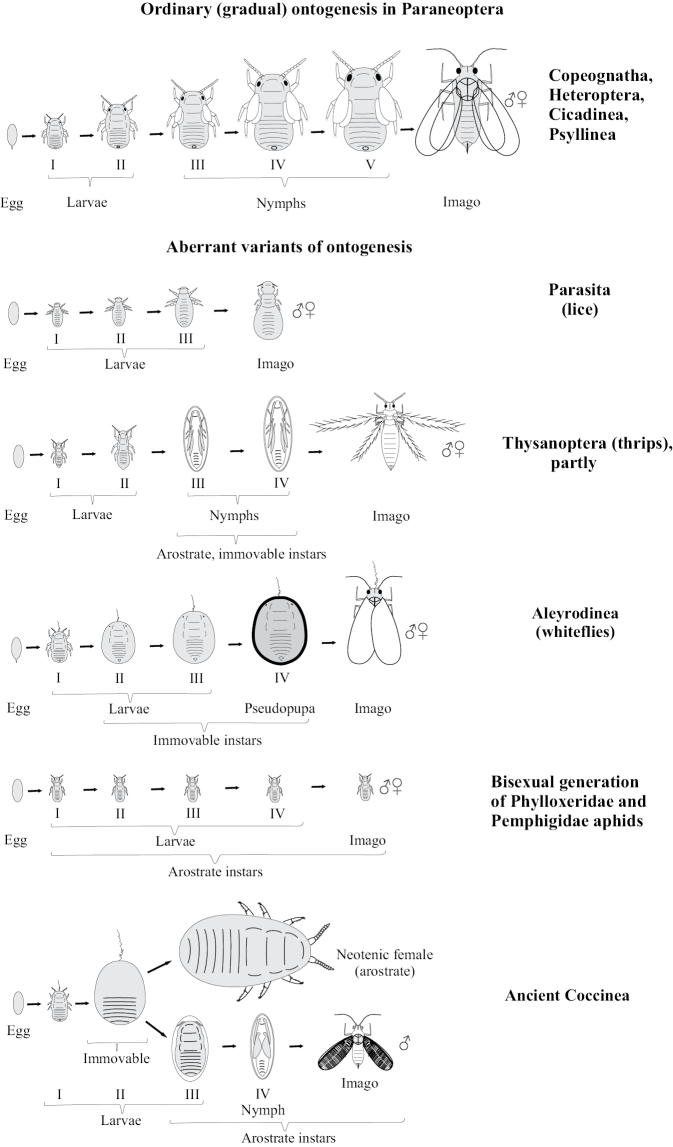
Ontogenesis in different groups of Paraneoptera.

**Figure 2.**
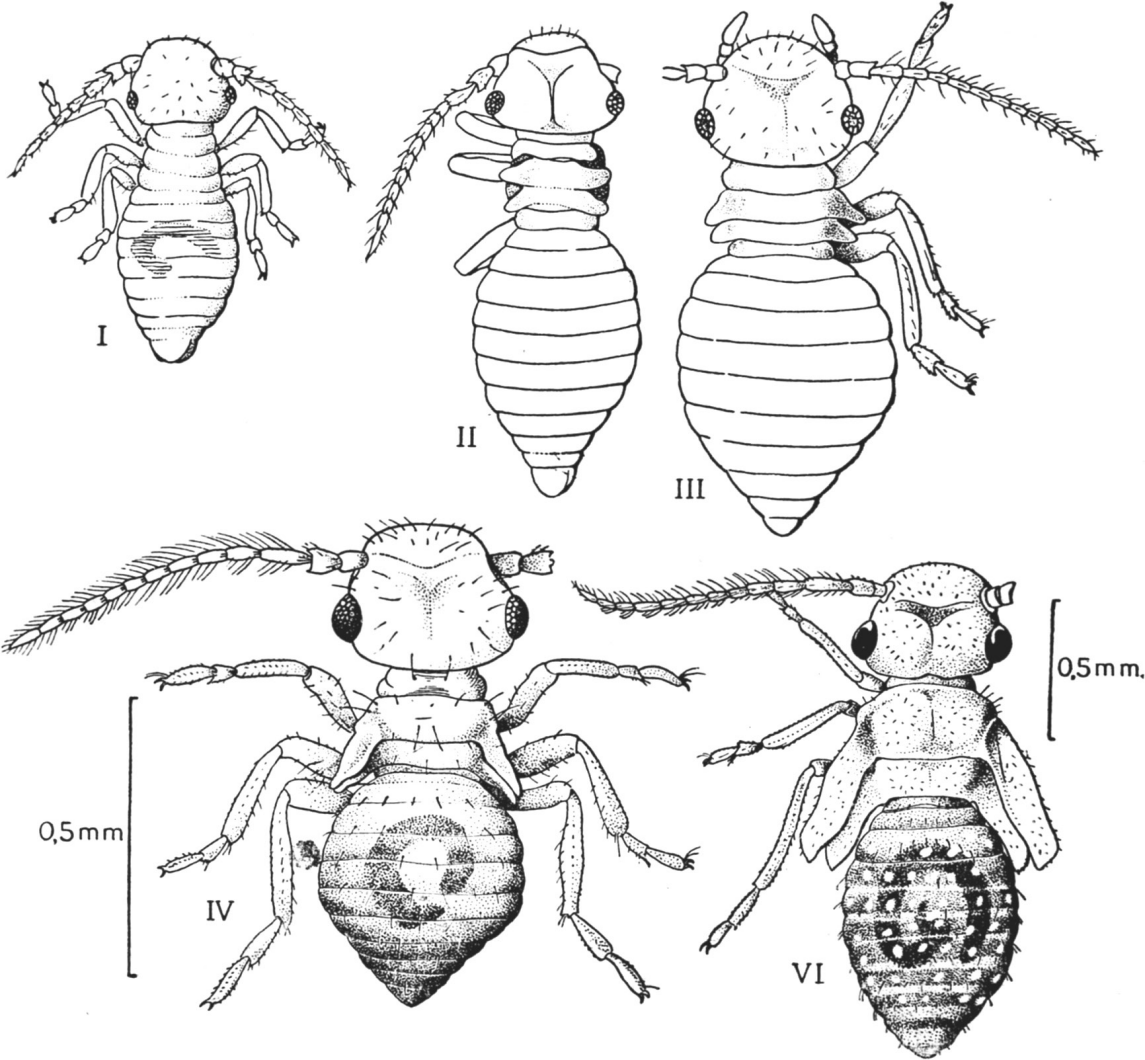
Larval instars of *Ectopsocusmeridionalis* Ribaga, 1904 (Copeognatha) (from Weber 1936). Larva V has a similar habitus with larva IV and is not figured.

The normal number of larval instars in Copeognatha is six, but in some rare cases this number can decrease to five, four or even three, this being associated with neoteny and alary polymorphism in the corresponding species (New and Lienhard 2007: 20–21, 113). One further interesting aberration of Copeognatha development is known in the European species *Prionoglarisstygia* Enderlein, 1909 (Prionoglarididae), which demonstrates a change of the initial type of the buccal apparatus to another type in course of the preimaginal ontogenesis (Ball 1936).

The small group Parasita (Mallophaga+Siphunculata+Rhyncophthirina), which originated from Copeognatha, is characterized by simplified ontogenesis with only 3 immature instars and a total lack of the protoptera and wings (Séguy 1951; Kluge 2020).

Psyllinea, Cicadinea, Heteroptera, and Coleorrhyncha generally retain the archaic “hemimetabolous” mode of the development and life cycle (Figs 1, 3), which may be monovoltine or polyvoltine, depending on species and climatic conditions, as in many other insects. Minute aberrations are connected with an unusual prolongaltion of the larval stage of the development (as in the family Cicadidae) or with a rear reduction of the number of the larval instars (as in Coleorrhyncha and in some species of Heteroptera). Thus, the periodical cicadas of the genus *Magicicada* Davis, 1925 show 13- or 17-year life cycles in different species with the duration of the imaginal instar 4–6 weeks only (Williams and Simon 1995). The decrease of the number of larval instars (from usual five to four) is known in Cicadinea for some brachypterous species of the tribe Almanini, family Dictyopharidae (Emeljanov 1980). The reduction of the number of the larval instars to four was noted in sporadic species of Heteroptera from the families Veliidae, Mesoveliidae, Nepidae, Nabidae, Anthocoridae, Cimicidae, Microphysidae, Miridae, Tingidae, Reduviidae, Tessaratomidae, while the vast majority of true bugs have five larval instars (see for review: Štys and Davidova-Vilimova 1989). The parasitic true bug family Polyctenidae, which is characterized by viviparity and paedogenesis, shows only three larval instars (Hagan 1951: 396; Maa 1959; Štys and Davidova-Vilimova 1989). All these instars have protoptera, which probably testifies the loss of two first larval instars in such ontogenesis (Štys and Davidova-Vilimova 1989). On the other hand, 6 larval instars are known only in several species of Miridae and Piesmatidae true bugs, which demonstrate variation in number of immature instars from 4 to 6 (Štys and Davidova-Vilimova 1989).

**Figure 3.**
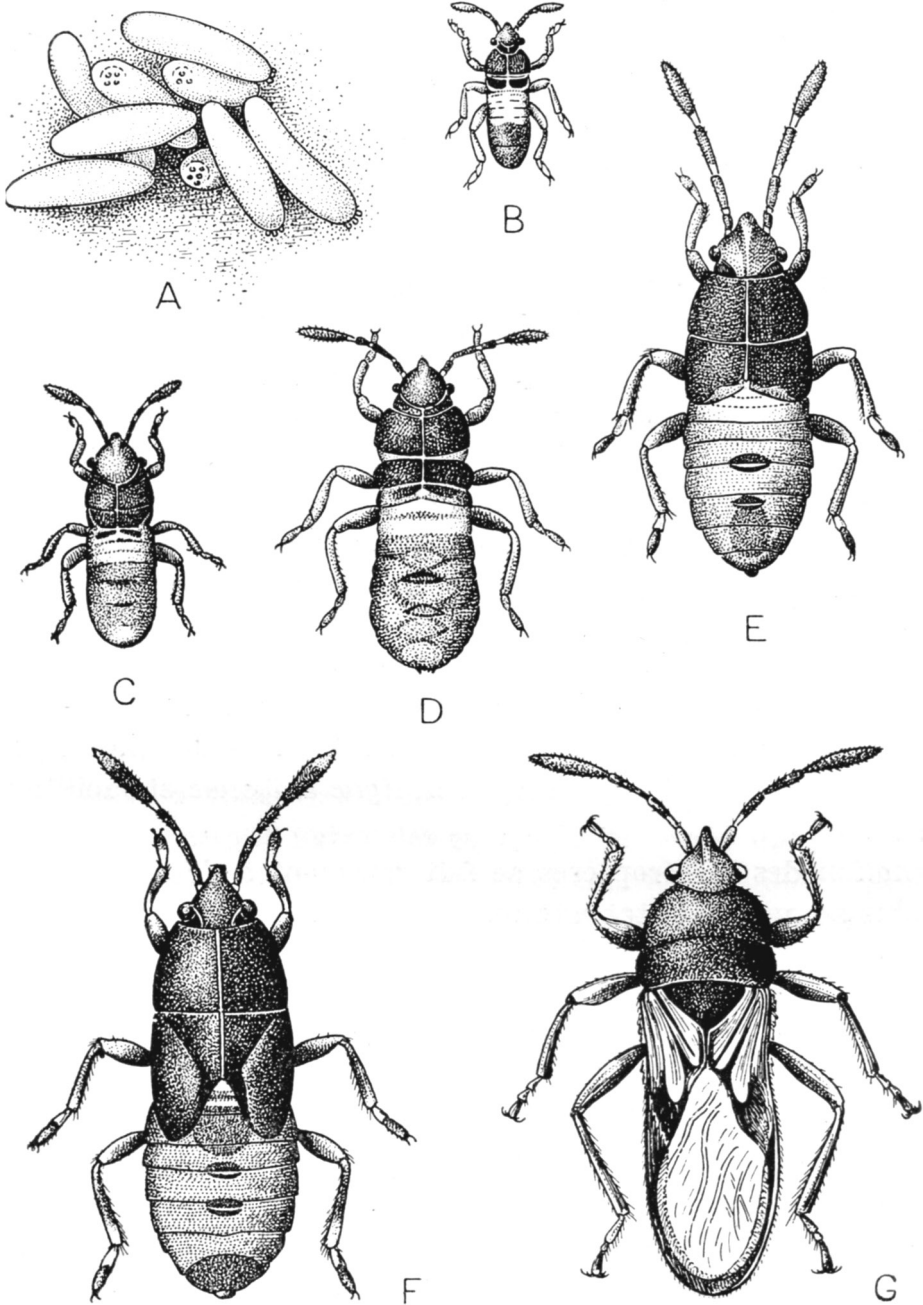
Ontogenesis of *Blissusleucopterus* (Say, 1832) (Heteroptera) (from Packard and Benton 1937) **A** eggs **B–F** larval instars **G** imago.

Species of the small relict order Coleorrhyncha have only 4 larval instars (China 1962; Evans 1981).

Comparatively small groups of Paraneoptera, such as thrips (Thysanoptera), lice (Parasita), whiteflies (Aleyrodinea), scale insects (Coccinea) and aphids (Aphidinea) show various curious aberrations in the postembryonic development (Fig. 1). In contrast to other Paraneoptera, thrips (Thysanoptera), whiteflies (Aleyrodinea) and scale insects (Coccinea) have ontogenesis with one or several immobile instars. Thus, ontogenesis of thrips (Thysanoptera) shows various patterns in different families, but their most primitive ontogenesis includes two first mobile larval instars in both sexes, two quiescent starving nymphs with partly reduced mouthparts and a mobile imago with normally developed legs, antennae, wings and mouthparts (Figs 1, 4) (Pesson 1951; Kluge 2020).

**Figure 4.**
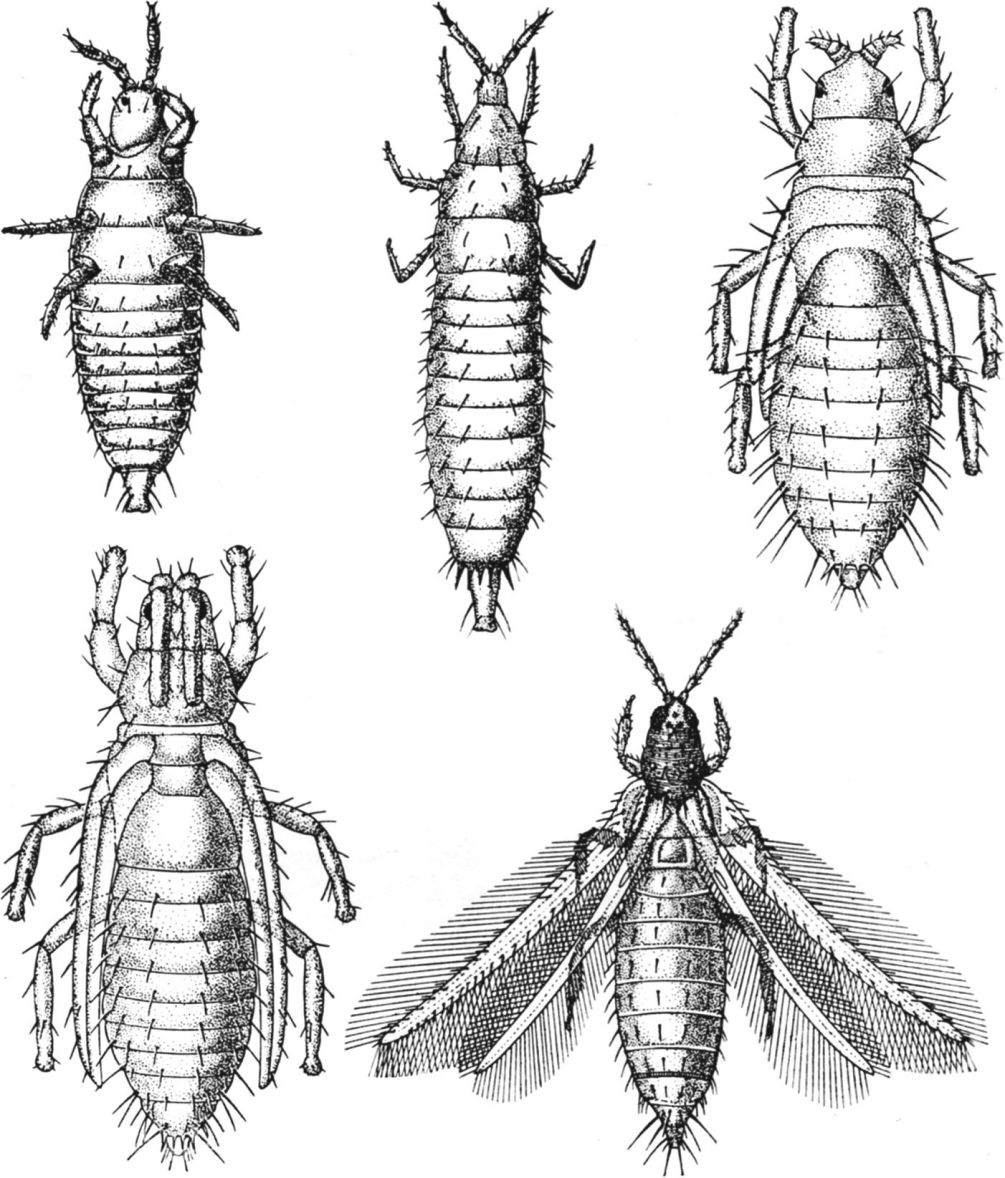
Ontogenesis of *Taeniothripsinconsequens* (Uzel, 1895) (Thysanoptera) (from Cameron and Trenerne 1918).

In whiteflies, the larva in all known species loses mobility after the first molt, and next three larval instars have only vestigial legs and are absolutely immobile (Fig. 1); moreover, all immature stages do not have protoptera; the ultimolarva (pseudopuparium) additionally is able to survive a long period of starvation. The pseudopuparium molts into the imago of both sexes which have well developed legs, antennae and wings. This ontogenesis is in fact similar to the metamorphosis of the holometabolous insects and is the most aberrant not only amongst Paraneoptera, but of Insecta as a whole.

In scale insects (Coccinea) two preadult instars of male are quiescent (arostrate and with non-segmented appendages). Such instars are in fact analogous to pupal instars of Holometabola (Gabritschesky 1923; Zakhvatkin 1975; Gavrilov-Zimin 2018). When such instars have protoptera they can be named as quiescent nymphs. Adult males of all scale insects are arostrate, but usually have normally developed legs and wings. In the female life cycle of all scale insects the normal imaginal stage is absent and larva of third of forth stage (neotenic female) is able to copulate with adult male and reproduce progeny. In some archaeococcids of the family Margarodidae s.l. (subfamilies Margarodinae s.s., Xylococcinae, Callipappinae) the second and third (if present) female instars are apodous, but actively suck sap from its host plant, whereas the neotenous female is mobile, has legs, but is arostrate (Fig. 5). On the other hand, most other archaeococcids (Margarodidae: Monophlebinae, Ortheziidae, Phenacoleachiidae, Carayonemidae) and many neococcids (superfamily Coccoidea) have simple direct ontogenesis of females, with all stages mobile (Fig. 6). Such neococcids as Aclerdidae, Asterolecaniidae s.l., Keriidae, Beesoniidae, Phoenicococcidae, Diaspididae, along with some species and genera of Pseudococcidae, Eriococcidae and Coccidae lose their legs during the first or last molt of females without alternation of movable/immovable instars (Fig. 7).

**Figure 5.**
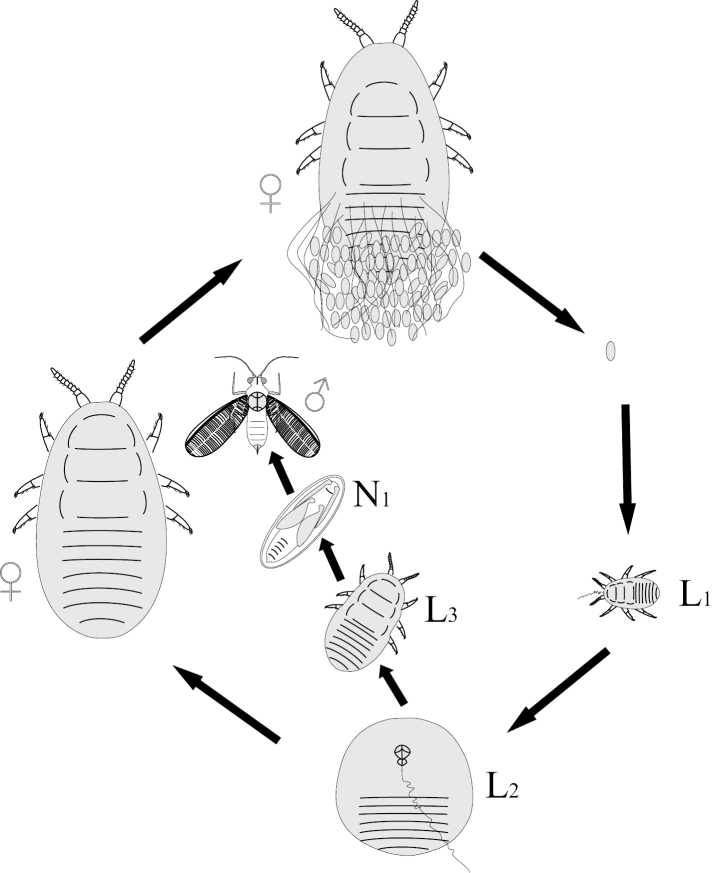
The archaic cycle of Matsucoccini (Coccinea) pattern. L – larva; N – nymph.

**Figure 6.**
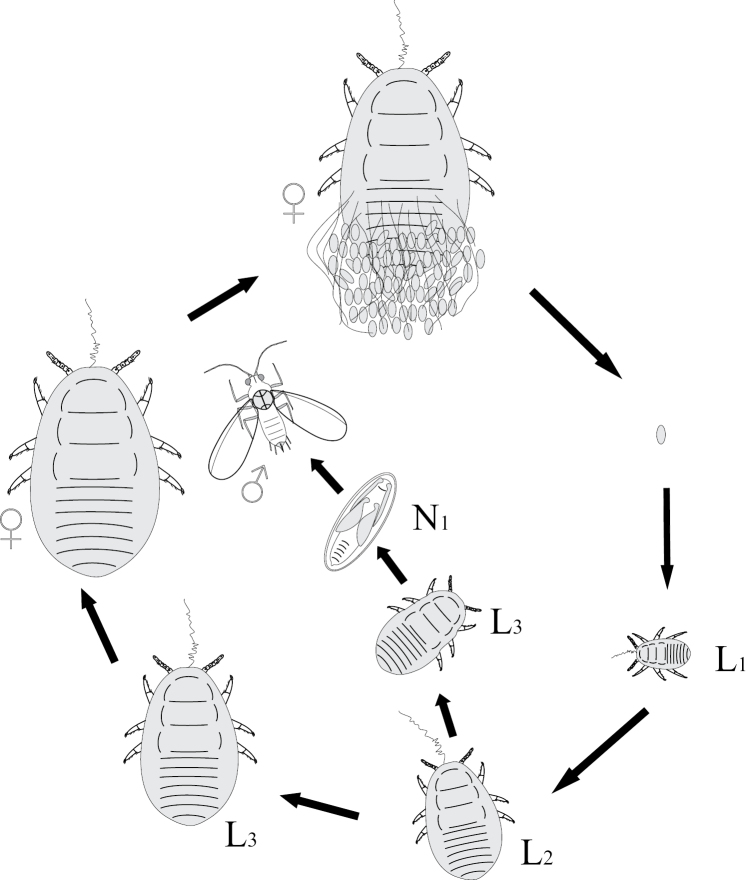
Life cycle of Monophlebinae (Coccinea) pattern.

**Figure 7.**
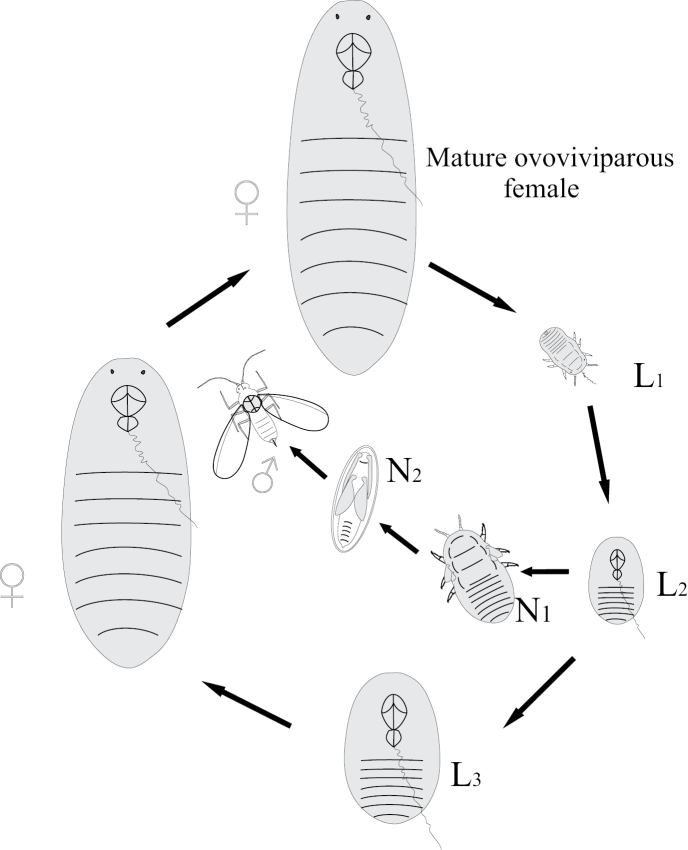
Life cycle of apodal neococcids (Coccoidea) pattern.

Borchsenius (1956) presumed that the original ontogenesis of Coccinea was similar to that of whiteflies (Aleyrodinea), i.e. apodal stages were present in both female and male ontogenesis. However, this presumption was not supported by any detail argumentation or comparative analysis of the life cycles of different scale insects and other Paraneoptera. The opposite hypothesis was provided and comprehensively argued by Danzig (1980). She supposed that the ancestor of all scale insects had a simple direct ontogenesis similar to that of Psyllinea, Cicadinea and Heteroptera. Then in course of the evolution of Coccinea the ontogenesis became more complete in males only, whereas females retained the direct cycle, but lost the winged imago (neoteny). In the frame of such approach the alteration of mobile/immobile stages and the aphagia of adult females in some Margarodidae s.l. was considered as a collateral evolutionary occurrence. Recently, two modern investigations provided important new data for understanding the evolution of scale insect ontogenesis and as a result Borchsenius’ (1956) idea starts to seem more reliable. Firstly, Kluge (2010) studied several species of scale insects from different families (*Ortheziaurticae* Linnaeus, 1758, *Icerya* sp. and *Coccushesperidum* Linnaeus, 1758) and discovered paradoxical transformation of legs and antennae in the course of molts of these species from one larval instar to another (see below). Secondly, Gavrilov-Zimin (2018) comprehensively analyzed the data on life cycles of all studied archeococcids in combination with comparative morphological analysis of all families, subfamilies and tribes of Orthezioidea. Both studies evidenced that the complicated ontogenesis with the alternation of mobile/immobile stages and with the arostrate imago of both sexes was initial in scale insect evolution and such ontogenesis may be considered as an apomorphy of suborder Coccinea.

Three scale insect species (from the families Ortheziidae, Margarodidae and Coccidae), studied by Kluge (2010), do not have any apodous stages in the female life cycle, but are characterized with a unique transformation of legs and antennae in course of the molt of one larval instar to another. Most of the internal soft tissues of every appendage, including the majority of muscles, degenerate before the molt and then emerge anew (Fig. 8). Moreover, the proximal segment of each appendage (coxa and scapus) newly grows in an unusual inverted position and everts only during ecdysis. As a result, the larva cannot move during the molt. This phenomenon occurs during all molts in female life cycle and during two first molts in male life cycle, whereas subsequent male molts (nymph I to nymph II and nymph II to adult male) are implemented without degeneration as in most other insects.

**Figure 8.**
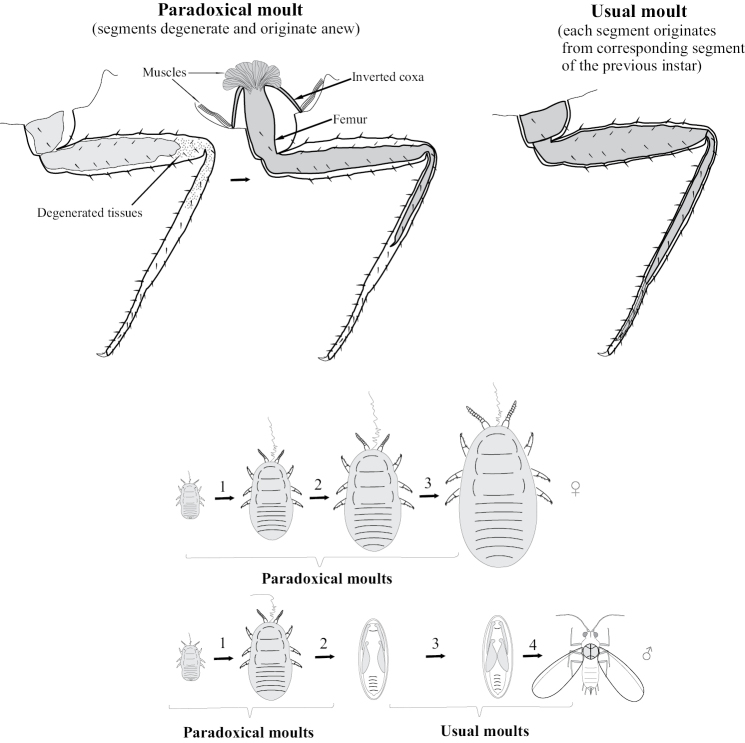
Simplified scheme of paradoxical and usual molts in scale insects. 1, 2, 3, 4 – molts from one instar to another.

As it was noted above, the ontogenesis with arostrate imago and apodal larvae of both sexes is considered now as a most primitive in scale insect evolution (Gavrilov-Zimin 2018). The appearance of such ontogenesis as well as all other variants of complicated metamorphosis, including holometabolism, was probably connected with the development of the larval instars in narrow shelters under the high pressure of unspecialized predators which decreases the number of openly lived insects in the late Paleozoic and Mesozoic biotopes (Rasnitsyn 1980). An ancestral group for both aphids and scale insect, as well as for all other Homoptera, was extinct Archescytinoidea (Popov 1980; Shcherbakov and Popov 2002). The Archescytinoidea lived in Permian geologic period (late Paleozoic Era) and were trophically connected with Gymnospermae trees. Females of Archescytinoidea laid eggs in unripe strobili of Gymnosperms and larvae dwelt there until ripe strobilus would dehisce (Popov 1980; Shcherbakov and Popov 2002). Such mode of life exactly permitted to protect immature stages from the predators. The sedentary life in strobili and then in cracks of tree bark was probably led to more and more significant difference between the larval instars and imago. As a result larvae of scale insect reduced and lost legs, and such apodous instars started to occupy most of the time of the life cycle. Additionally, in the condition of immobility the apodal body was probably more protected from entomopathogenic fungi, being evenly covered with wax. In contrast, unprotected imago started to be a short-lived instar with reduced mouthparts. Significant morphological contrast between the immobile apodous larva and highly mobile imago led to the appearance of the quiescent nymphal stages and so to the complicated metamorphosis (Fig. 9) – see for more details Gavrilov-Zimin (2018: 54–56).

**Figure 9.**
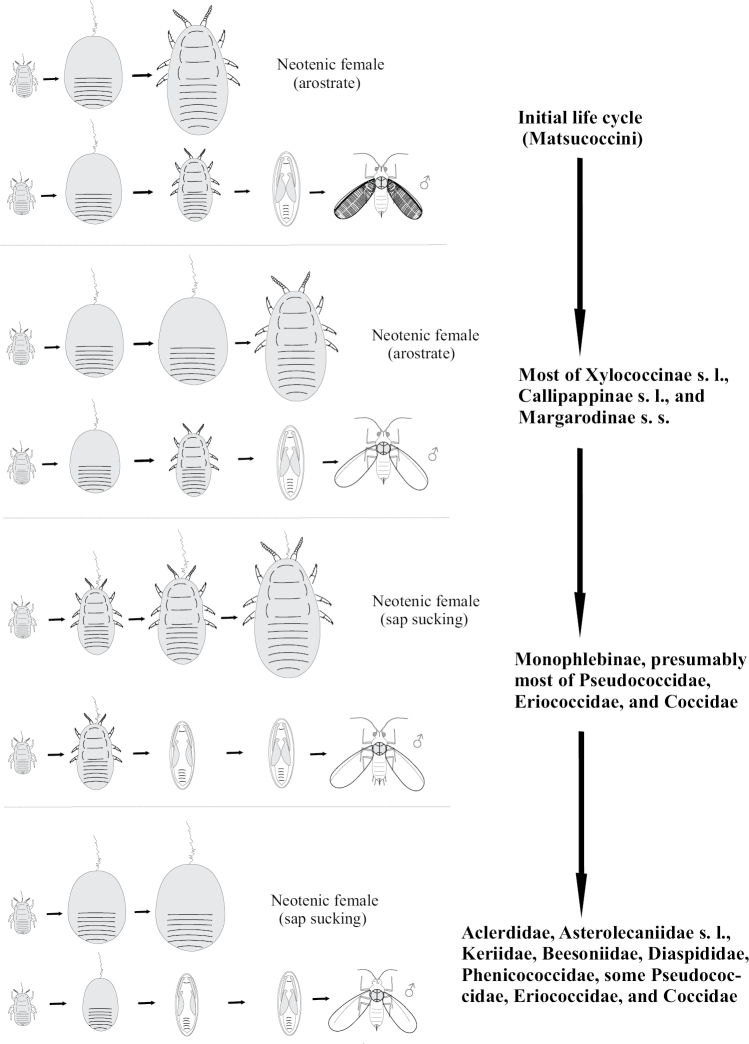
The evolution of ontogenesis in different groups of scale insects.

In some groups of scale insects, the female remains to live and reproduce inside the cuticle of the ultimolarva (Fig. 10). Such remarkable ontogenesis is known in some archeococcids of the subfamilies Callipappinae and Margarodinae (Morrison 1928; Vayssiére and Hughes-Schrader 1948; Morales 1991; Foldi 2005; Foldi and Gullan 2014; Gavrilov-Zimin 2018) and in some neococcids: in several genera of Phoenicococcidae s.l. (Stickney 1934), in about 60 genera of Diaspididae (Howell and Tippins 1990; Danzig 1993), in occasional species of Beesoniidae (Takagi 1992) and Eriococcidae (Gullan and Williams 2016). Thus, in the following genera of the tribe Cryptokermesini (Callipappinae): *Cryptokermes* Hempel, 1900, *Paracoelostoma* Morrison, 1927 and *Ultracoelostoma* Cockerell, 1902 the secundolarvae of both sexes secrete a resinous protective test that enlarges during subsequent development of the insect. The tertiolarva and neotenic female remain inside this test and moreover, inside the exuviae of the previous instar. Such instars are often considered as “pupillarial” (see, for example, Danzig 1993; Foldi and Gullan 2014; Gullan and Williams 2016) but this is incorrect, because the true puparium is the cover of the pupa, whereas scale insect females never have pupal instars in their ontogenesis. Gavrilov-Zimin (2018: 20, 59) introduced the new term “exuviatrium” for the larval exuvium which is used by the next larva-like instar (including neotenic female) as a shelter. Correspondingly, the species with such a peculiarity may be named “exuviatrial”. In the genus *Mimosicerya* Cockerell, 1902 (also Cryptokermesini) female instars do not secrete any protective test, but the adult female is also exuviatrial, because it lives and lays eggs inside the strongly sclerotized ultimolarval exuvium. It seems rather obvious that such a mode of ontogenesis originated several times in the evolution of scale insects. Ontogenesis of Cryptokermesini probably originated from the archaic ontogenesis of Coelostomidiini ancestors, which is proved by the absence of mouthparts in the neotenic female and/or by the presence immobile stages of the ontogenesis. On the other hand, the ontogenesis of different exuviatrial neococcids (some of Eriococcidae, Phoenicococcidae s.l., Diaspididae and Beesoniidae) clearly originated from advanced pattern of ontogenesis of Monophlebinae-Pseudococcidae, since in all mentioned families the adult females are sap sucking (Gavrilov-Zimin 2018).

**Figure 10.**
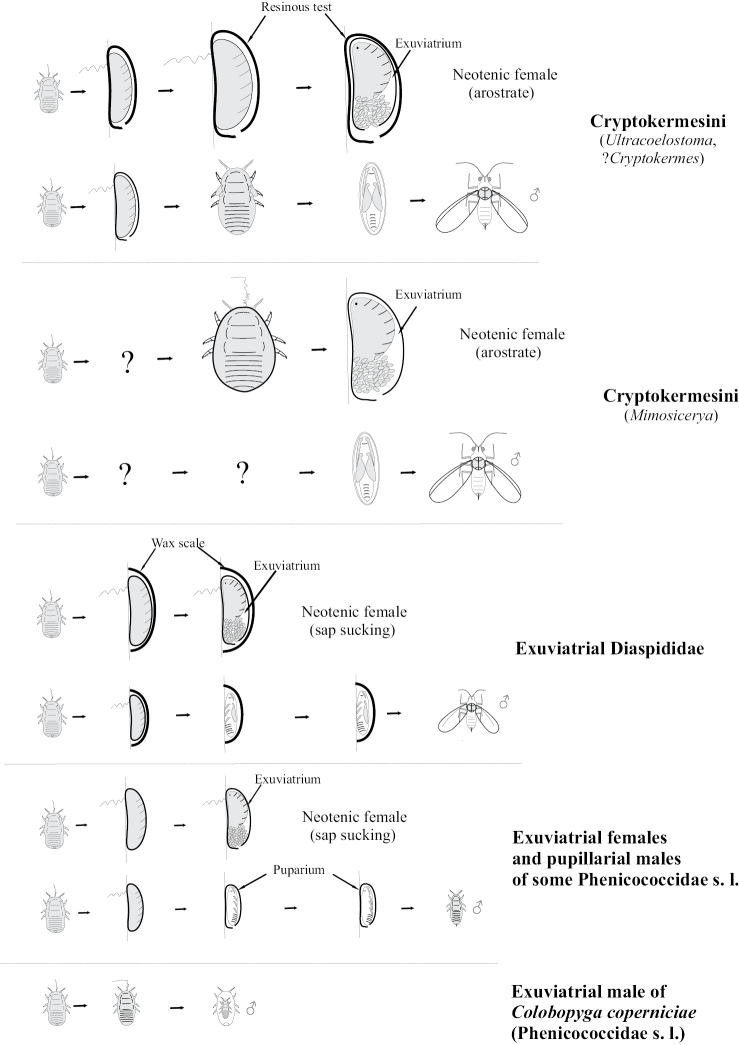
Exuviatrial and pupillarial instars in ontogenesis of different scale insects.

The true pupillarial development is now known in some scale insects of the family Phoenicococcidae s.l. only, in which quiescent male instars molt inside the exuvium of the secundolarva (Stickney 1934) (Fig. 10). On the other hand, the other species of this family, for example, *Colobopygacoperniciae* Ferris, 1952, are characterized by dwarfish apterous exuviatrial neotenic males, having only 2 immature instars (Köhler 1987).

Mating of scale insect winged males with apterous larva-like females and parthenogenetic reproduction of lava-like females are usually considered as examples of neoteny and paedogenesis, starting probably from the papers of Börner (1910) and Gabritschevsky (1923). This approach is based on the comparison of female and male ontogenesis and the presence of more numerous male instars in contrast to female ones in the life cycle: female has only 3–4 instars, all of which are always larva-like, whereas male has 5 instars, one or two of which are quiescent nymphs (with protoptera) and one is the alate male imago (Fig. 11). Moreover some species from different scale insect families (as in archaeococcids as well as in neococcids) show obligate or facultative presence of larva-like males (Fig. 11) (see for review Gavrilov-Zimin 2018). In case of facultative appearance of larva-like males they are present in the population together with the normal alate males which undergo complicated individual development, including 2–3 larval and 1–2 quiescent nymphal instars. It was clearly demonstrated in some species that the apterous males have fewer instars than alate males – three or even two immature instars instead of four (Hadzybeyli 1958, 1969; Hafez and Salama 1967; Köhler 1987).

**Figure 11.**
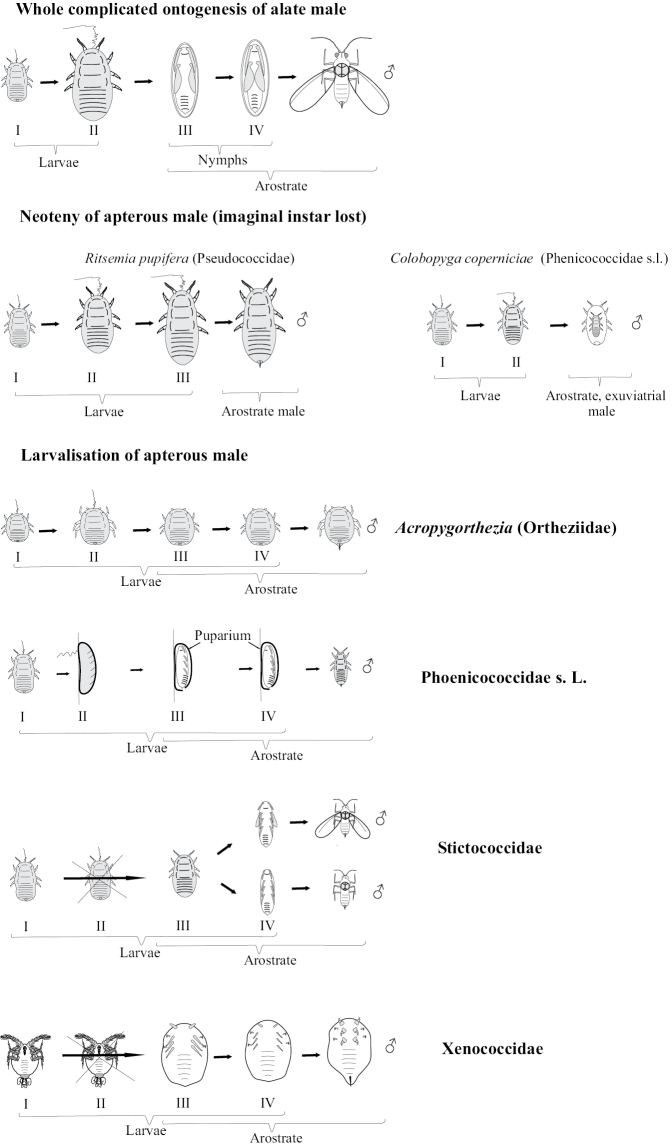
Neoteny and larvalization of males in different families of scale insects (Coccinea).

On the other hand, the apterous males of Phoenicococcidae s.l. (according to Stickney 1934), Xenococcidae (according to Williams 1986, 1998 and Kishimoto-Yamada et al. 2005), *Acropygorthezia* (according to LaPolla et al. 2008), and *Putosuperbus* (Leonardi, 1907) (according to Gavrilov-Zimin 2018) have the same number of quiescent arostarte instar(s) before molting into apterous males.

In Stictococcidae according to Richard (1971) both apterous and alate males have four instars, which is less than the usual number (five) in alate males of other studied scale insects. Moreover, the loss of mouthparts in Stictococcidae males occurs during the first molt. This fact may be considered as an evolutionary loss of the second feeding larva in the ontogenesis (Fig. 11).

Borchsenius (1956) disputed the neoteny in scale insects and explained the evolution of coccid ontogenesis in the frame of “larvalization” of both females and apterous males. He supposed that the evolutionary reduction of the general number of instars was connected with the loss of quiescent instars, but not with the loss of imaginal instar itself. This idea is contradicted by the following facts: 1) All cytogenetically studied scale insect males have spermatogonial meiosis in third instar, whereas fourth instar and adult male have fully developed sperm bundles in their testes; the oogenesis also occurs in third instar of female and so, this instar may be clearly considered as a reproduced neotenic tertiolarva. 2) The real imaginal larvalization with the absence of nymphs may be observed in aphids, sister group to scale insects. Apterous larva-like females and males of aphids (excluding *Stomaphis* Walker, 1870 discussed below) usually have the same number of instars (five) in their ontogenesis as alate females of the same population (Fig. 15). So, the true larvalization is not connected with the reduction of the number of instars, but with their modification only. In this meaning, the term “larvalization” may be used at least for apterous males of *Acropygorthezia* (Ortheziidae), Xenococcidae, Phoenicococcidae, and Stictococcidae, which save quiescent preadult instars in their ontogenesis (Fig. 11).

In many scale insects, for example, in such archeococcids as *Gueriniella* Fernald, 1903 or different species of *Icerya* Signoret, 1876, and in numerous species of neococcids from different families, males are unknown and probably completely absent. In these cases, the female tertiolarva reproduces in a parthenogenetic or hermaphroditic way and so can be considered as a paedogenetic female.

The ontogenesis of aphids (Aphidinea), usually consisting of 6 instars (egg, 4 larval instars and imago) (Figs 15, 16), is complicated in most cases by cyclic parthenogenesis with a regular alternation of bisexual and parthenogenetic generations and with or without a regular alternation of the host plants (Mordvilko 1914, 1934, 1935; Pesson 1951; Popova 1967; Blackman 1987; Moran 1992). In the archaic aphid superfamily Phylloxeroidea, both parthenogenetic and bisexual generations lay eggs (Fig. 12), whereas in the “advanced” superfamily Aphidoidea parthenogenetic females produce offspring by placental viviparity or ovoviviparity (see also the second paper (chapter) in the present Issue). In so-called “holocyclic” aphids the life cycle includes: 1) a bisexual generation (wingless larva-like or alate females and males in different families of aphids), which copulates and produces eggs; 2) a generation of wingless (rarely alate) females “fundatrices”, which hatch from the eggs and then produce next generation by parthenogenesis; 3) several or many wingless parthenogenetic generations of females (“virginoparae”); 4) a generation of alate females which may or may not migrate to another host plant, another part of the same plant (monoecious cycle) or another species of host plant (dioecious cycle) and then give rise a new generation by parthenogenesis; 5) several or many wingless or alate parthenogenetic generations of females on the same or on the secondary host plant; 6) a generation of alate females, so-called “sexuparae” which migrate back to primary host plant (in dioecious cycle) and parthenogenetically produce the bisexual generation or “gynoparae”, which produce only females or “androparae”, which produce only males (Fig. 13). In the whole cycle (Fig. 16) the wintering egg gives rise to fundatrix female, which produce virginoparae and/or migrant females by thelytokous parthenogenesis during the summer time; in the autumn the generation of sexuparae females appears and produce females and males by deuterotokous parthenogenesis; these sexual instars copulate and the females lay overwintering eggs. In course of the development of the parthenogenetic egg only one maturation division of meiosis takes place without reduction of the diploid number of chromosomes and with a throwing out one polar body; however, the crossing over of the homologous chromosomes probably occurs in early prophase (Blackman 1987: 177). In sexuparae females the oocytes destined to produce male, the X chromosomes form a bivalent with two homologues joined end-to-end (Fig. 16), and then one of X-chromosome degenerates, whereas the other one divides equationally with the autosomes (Orlando 1974; Blackman 1987: 172). During male meiosis one of the secondary spermatocytes gets an X-chromosome and more cytoplasm than the other spermatocyte, which degenerates, which is a unique feature of the Aphidoid genetic system (Blackman 1987; Gavrilov-Zimin et al. 2015).

**Figure 12.**
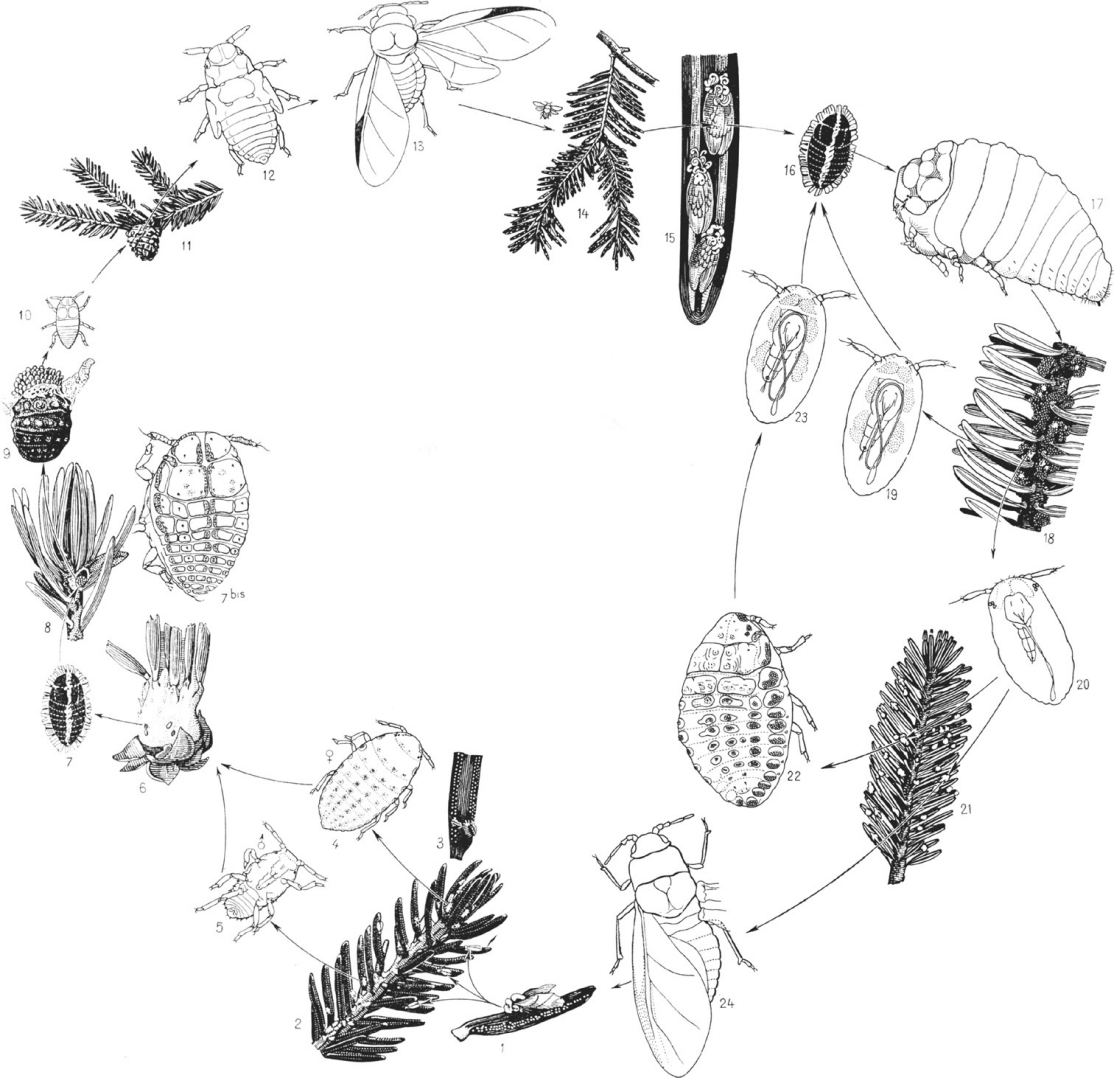
Biennial life cycle of *Adelgesnordmannianae* (Eckstein, 1890) (Aphidinea), from Pesson, 1951, with changes. Stages **1–13** occur during first year on *Piceaorientalis* (Linnaeus, 1763): **1** female “sexupara”, migrated from fir (June) **2–3** larval instars on spruce (July) **4–5** female and male (July) **6** oviposition (July) **7** wintering larva (August-March) **7^bis^–8** female “fundatrix” (April) **9** oviposition (April) **10–11** larva, producing a gall on twig of spruce (Mai) **12** nymph (June) **13** migrating female (July). Stages **14–24** occur during second year on *Abiesnordmanniana* (Steven, 1838): **14–15** females, migrating from spruce lay eggs (July) **16** overwintering larva (August-April) **17–18** parthenogenetic female and its oviposition (Mai) **19–23** new parthenogenetic generations (Mai-June) **24** alate female, migrating to spruce (June).

**Figure 13.**
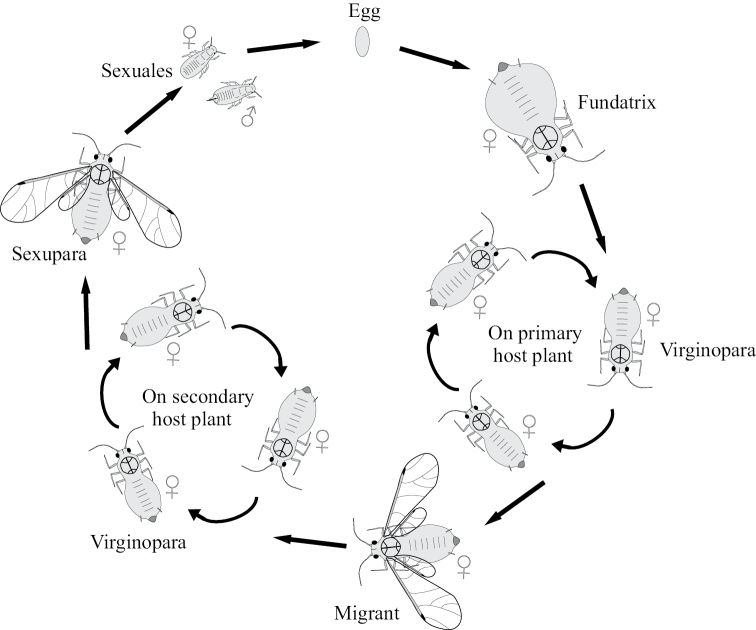
Generalized scheme of the annual cycle of generations in holocyclic aphids with wingless “virginoparae” and wingless larva-like “sexuales”.

Some species of aphids, especially in tropical climates, demonstrate a simplified (“anholocyclic”) life cycle with only parthenogenetic generations and without regular changing of the host plants. Often the number of parthenogenetic generations may be 15–20 per year and sometimes up to 40 per year (Mordvilko 1934: 37; Gullan and Martin 2009). On the other hand, in some aphid species from the families Greenideidae and Aphididae the annual cycle may be reduced (in some parts of the species range) to only two generations: bisexual generation give rise a generation of fundatrices, which parthenogenetically produces new bisexual generation (Fig. 14) (Takahashi 1959; Strathdee and Bale 1995; Stekolshchikov and Khruleva 2014).

**Figure 14.**
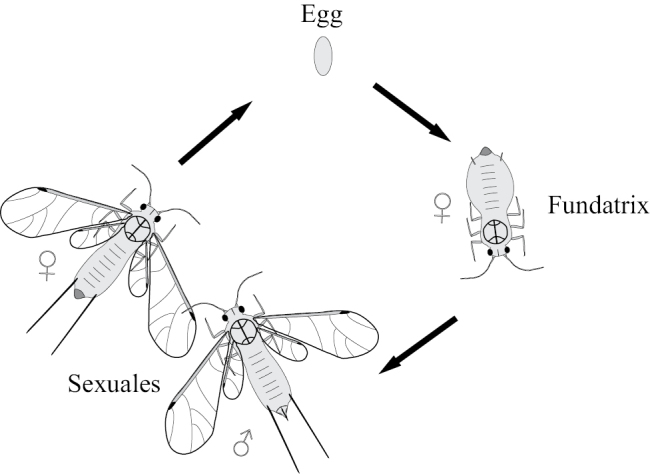
Generalized scheme of the simplified annual cycle of some species of Greenideidae (Aphidinea).

**Figure 15.**
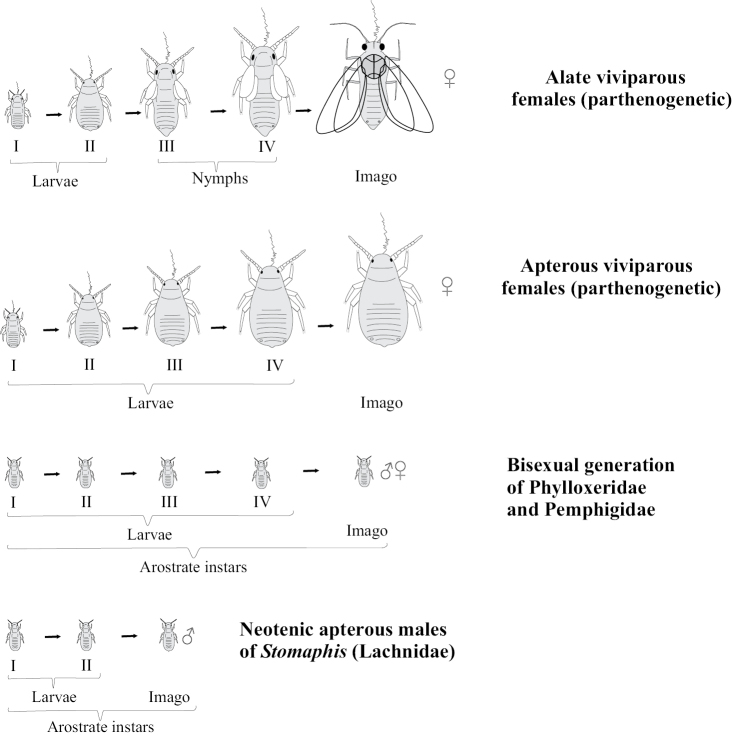
Ontogenesis and larvalization of aphids (Aphidinea).

**Figure 16.**
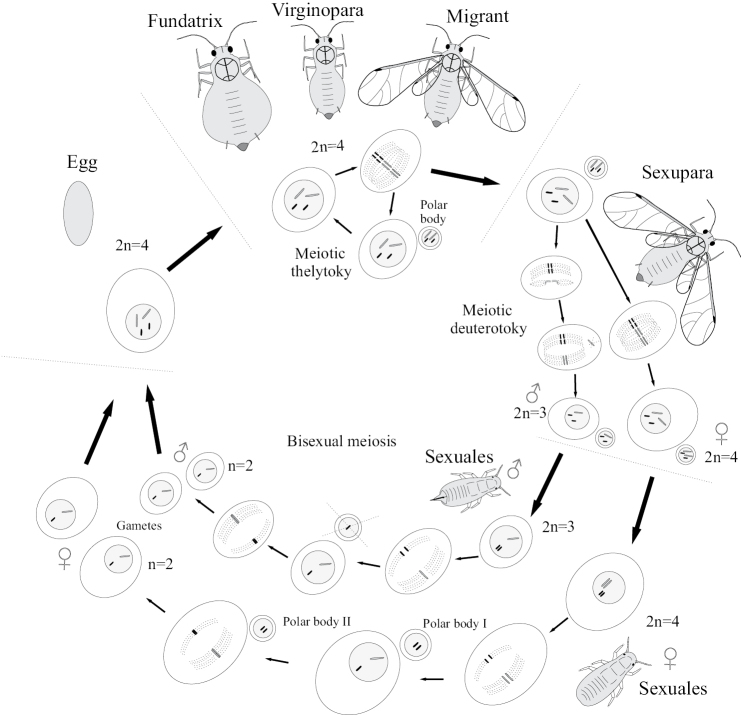
Cyclic parthenogenesis and its cytogenetic mechanisms in holocyclic aphids with diploid chromosome number 4.

The arostrate instars in aphids are known as a whole bisexual arostrate generation in all species of Phylloxeridae and Pemphigidae (Mordvilko 1914; Popova 1967). All four larval instars and imago (of both sexes) in this generation do not have mouthparts and do not increase the body size during molts (Fig. 1). In the aphid genus *Stomaphis* Walker, 1870 (Lachnidae) only neotenic males are arostrate, whereas all female instars have well developed mouthparts (Mamontova 2008, 2012; Depa et al. 2015). The species of this genus save only two or three (instead of four) immature instars in ontogenesis (Fig. 5), that is considered as a clear example of male neoteny (Mamontova 2008, 2012; Depa et al. 2015).

There are no doubts that the ancestral ontogenesis and life cycle of aphids was based on obligatory bisexual reproduction as in most other insects and Paraneoptera in particular. The appearance of intricate aphid cycles with an obligate alternation of bisexuality and parthenogenesis was connected with the original adaptation of the group to the temperate climate of the Holarctic (Mordvilko 1934: 47, 1935: 34), where an absolute majority of aphid species, including all archaic groups, are still found. On the contrary, aphids in the tropical zone of the world and in the Southern Hemisphere are comparatively rare and represented by some “advanced” families only. The evolutionary appearance of the aphid cyclic parthenogenesis, based on the unique “Aphidoid” genetic system, is considered as an apomorphic character of the suborder Aphidinea (Moran 1992; Gavrilov-Zimin et al. 2015). This genetic system excludes the reduction of the modern aphid life-cycle to only one bisexual generation (see also the next papers(chapters) of this Issue).

The overall picture of ontogenesis in Paraneoptera shows peculiar and even enigmatic evolutionary parallelisms – the independent appearance of the similar aberrations in related, but not sister phylogenetic lineages. Such parallelisms are also known in other fields of Paraneoptera biology – in morphology, anatomy, cytogenetics, reproductive biology, etc. (see for details: Gavrilov-Zimin et al. 2015; Gavrilov-Zimin 2020). Thus, the quiescent instars are present in ontogenesis of Thysanoptera and in two of five suborders of Homoptera: Coccinea and Aleyrodinea, which are not sister to each other according to the current interpretation of the phylogeny (see Fig. 17). The general reduction of the number of larval instars from 5–6 to 2–4 occurs in Parasita, Thysanoptera, Coleorrhyncha, Aphidinea, Aleyrodinea, Coccinea (especially in females) and also in occasional genera and families of Copeognatha and Heteroptera, whereas Psyllinea and Cicadinea show a rather high (5) and stable number of the larval instars. It seems that the reduction of the number of instars was associated with different causes in various groups of Paraneoptera. In some cases, the true imaginal instar disappears and the previous larval instars start to reproduce in course of neoteny or paedogenesis (as in aphids, scale insects and probably in some booklice and some true bugs). In lice the ontogenetic reduction probably connects with the loss of as true imaginal as well as of true first larval instars in view of the so-called embryonal molt. In whiteflies and thrips some intermediate larval instars were probably “merged” in one or two quiescent larval instars. However, the questions, connected with the clear interpretation of homology/non-homology of the instars in the ontogenesis of most Paraneoptera are presently rather controversial and very poorly studied.

**Figure 17.**
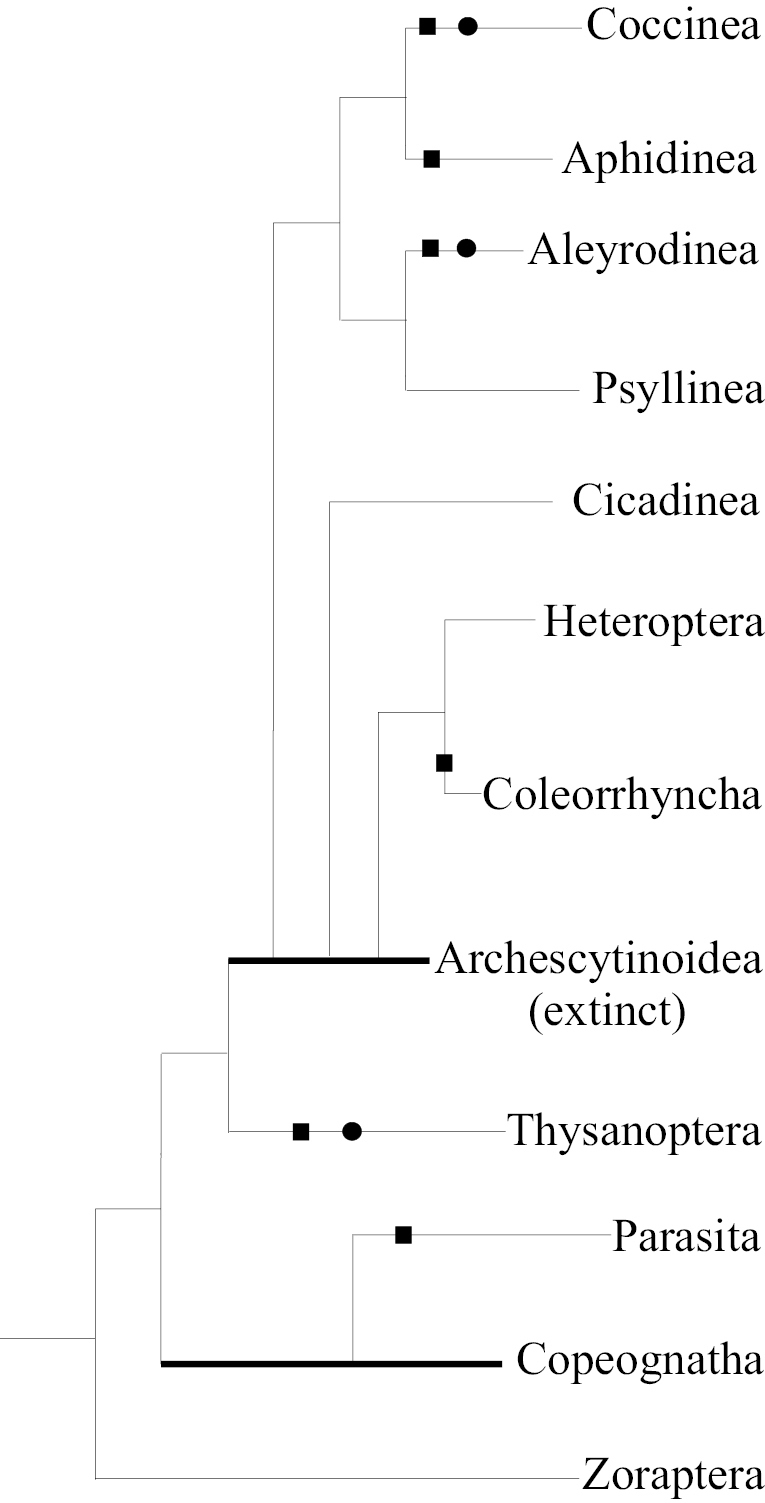
The phylogenetic tree of Paraneoptera based on Shcherbakov and Popov (2002), Kluge (2020), Gavrilov-Zimin (2020) with modifications. The phylogenetic lines with the quiescent larval instars in ontogenesis are indicated by black solid circles (●); the lines with general reduction of the number of larval instars are indicated by black solid squares (■). Bold lines are used for paraphyletic taxa.
